# The Quest for Molecular Regulation Underlying Unisexual Flower Development

**DOI:** 10.3389/fpls.2016.00160

**Published:** 2016-02-19

**Authors:** Rómulo Sobral, Helena G. Silva, Leonor Morais-Cecílio, Maria M. R. Costa

**Affiliations:** ^1^Biosystems and Integrative Sciences Institute, Plant Functional Biology Centre, University of MinhoBraga, Portugal; ^2^Departamento de Recursos Naturais Ambiente e Território, Linking Landscape, Environment, Agriculture and Food, Instituto Superior de Agronomia, Universidade de LisboaLisboa, Portugal

**Keywords:** *Quercus suber*, male and female flower development, unisexuality, monoecy, RNA-seq, transcriptomics

## Abstract

The understanding of the molecular mechanisms responsible for the making of a unisexual flower has been a long-standing quest in plant biology. Plants with male and female flowers can be divided mainly into two categories: dioecious and monoecious, and both sexual systems co-exist in nature in *ca* of 10% of the angiosperms. The establishment of male and female traits has been extensively described in a hermaphroditic flower and requires the interplay of networks, directly and indirectly related to the floral organ identity genes including hormonal regulators, transcription factors, microRNAs, and chromatin-modifying proteins. Recent transcriptomic studies have been uncovering the molecular processes underlying the establishment of unisexual flowers and there are many parallelisms between monoecious, dioecious, and hermaphroditic individuals. Here, we review the paper entitled “Comparative transcriptomic analysis of male and female flowers of monoecious *Quercus suber*” published in 2014 in the Frontiers of Plant Science (volume 5 |Article 599) and discussed it in the context of recent studies with other dioecious and monoecious plants that utilized high-throughput platforms to obtain transcriptomic profiles of male and female unisexual flowers. In some unisexual flowers, the developmental programs that control organ initiation fail and male or female organs do not form, whereas in other species, organ initiation and development occur but they abort or arrest during different species-specific stages of differentiation. Therefore, a direct comparison of the pathways responsible for the establishment of unisexual flowers in different species are likely to reveal conserved modules of gene regulatory hubs involved in stamen or carpel development, as well as differences that reflect the different stages of development in which male and/or female organ arrest or loss-of-function occurs.

## Introduction

Unisexuality is considered to be an important transition in the evolutionary history of angiosperms (Barrett, 2010). The emergence of separate male and female traits in the same individual (**monoecy**) or in different individuals (**dioecy**) has evolved many times in ca 10% of angiosperms species from a hermaphroditic ancestral state (Charlesworth and Charlesworth, 1978; Tanurdzic and Banks, 2004). Here, we present a focused review of our current understanding on the different mechanisms underlying unisexual flower development. We will focus our attention in high-throughput transcriptomic studies using unisexual flowering species, which is providing large amounts of information and may uncover the molecular mechanisms responsible for male and female unisexual flower differentiation and determination.

KEY CONCEPT 1 **Monoecy and Dioecy**

Two major unisexual systems occur in the angiosperms: **Dioecy**—sexual system in which individual plants have either male or female flowers. **Monoecy**—sexual system in which both male and female flowers coexist in the same individual.

## The making of male and female organs

In angiosperms, endogenous and environmental cues control highly specialized gene expression programmes that establish the male and female organs within a flower. These mechanism have been extensively studied in hermaphrodite flowers and led to the elaboration of the ABCDE model, in which each class of genes is recruited in the flower meristem to specify the identity of sepals, petals, stamens, carpels, and ovules (Bowman et al., 1989, 1993; Coen and Meyerowitz, 1991; Flanagan et al., 1996; Liljegren et al., 2000; Pelaz et al., 2000). In *Arabidopsis thaliana*, and in other species with hermaphrodite flowers, B combined with the C and E class genes specify stamen identity, whereas C and E genes, together, specify carpel identity (for a review see Alvarez-Buylla et al., 2010; Litt and Kramer, 2010; Bowman et al., 2012). Homologs for the ABCDE model genes have been identified and associated with the male and female organ differentiation in many hermaphrodite species in what seems to be a conserved molecular mechanism (Theissen and Melzer, 2007; Greenup et al., 2009; Andrés and Coupland, 2012; Bowman et al., 2012). These genes seem to be also playing a role in the differentiation of unisexual organs in dioecious and monoecious species (Kater et al., 2001; Sather et al., 2010).

The developmental mechanisms that lead to flowers with different sex may require differential redeployment of the ABCDE regulatory network. Differences in the regulation of upstream effectors of the homeotic regulators may originate flowers that fail to initiate female or male organ primordia being unisexual by inception—**Type II flowers** (Mitchell and Diggle, 2005). On the other hand, many flowers become unisexual after flower organs are specified, but during the process of differentiation, carpel or stamen abortion or arrest occur and the organs become non-functional (**Type I flowers**). There is however some difficulty in distinguish morphologically a true Type II flower, that fails to initiate the developmental programs of the undesired organ, from a Type I flower that suffers organ abortion or arrest at a very early stage of organ initiation and development (Mitchell and Diggle, 2005).

KEY CONCEPT 2 **Type I flowers, androecium, gynoecium, and Type II flowers**

Different pathways that originate gender dimorphism: **Type I flowers** are bisexual at initiation and become unisexual by termination of the development of the **androecium** (the male reproductive organs) or **gynoecium** (the female reproductive organs). In **Type II flowers**, sex differentiation occurs before stamen or carpel primordia initiation (Diggle et al., 2011).

**Figure 1:**
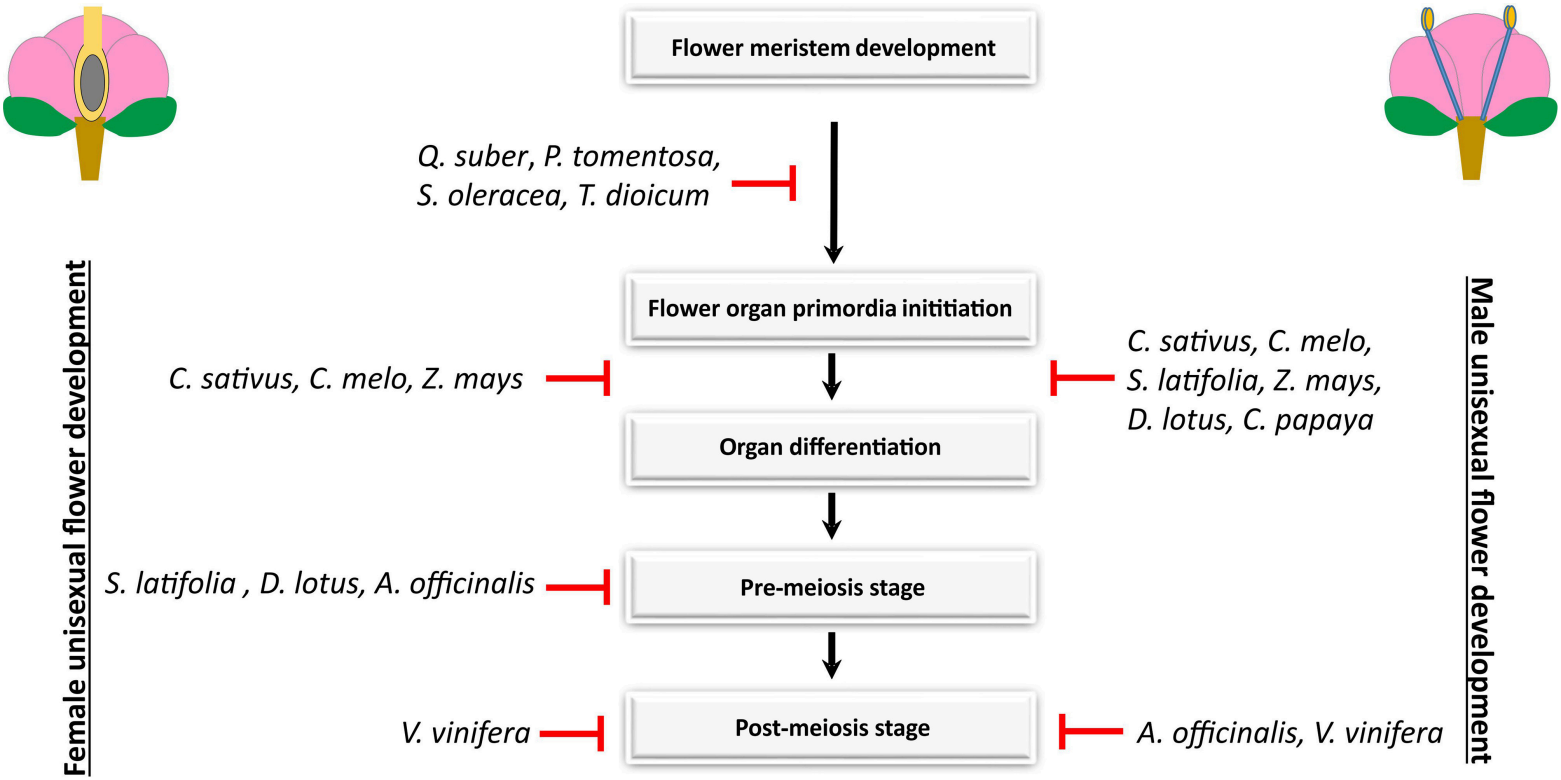
**Developmental path to unisexuality in male and female flowers in monoecious and dioecious species**. In *Quercus suber, Populus tomentosa, Spinacea oleracea*, and *Thalictrum dioicum*, sex differentiation occurs prior to flower organ primordia initiation (Varela and Valdiviesso, 1996; Boavida et al., 1999; Sheppard et al., 2000; Di Stilio et al., 2005; Pfent et al., 2005; Sather et al., 2010). In *Carica papaya*, the pistil degeneration is clear at early stages of organ development in male flowers but female flowers have no traces of stamens (Ronse Decraene and Smets, 1999). Stamen and pistil development in *Cucumis sativus, Cucumis melo*, and *Zea mays* arrests during early organogenesis (Le Roux and Kellogg, 1999; Bai et al., 2004; Boualem et al., 2008). The abortion of stamens in *Silene latifolia* and *Diospyros lotus* occurs later than the abortion of the pistil, at a pre-meiotic stage (Grant et al., 1994; Akagi et al., 2014). In *Asparagus officinalis*, the arrest of stamens occurs somewhat early than the arrest of pistils (Caporali et al., 1994). Stamen and pistil degeneration in *Vitis vinifera* occurs at the post-meiotic stage (Caporali et al., 2003).

The extensive utilization of forward and reverse genetics in *A. thaliana*, with more recent approaches such as microarrays, ChIPseq, and RNA-seq, led to the identification of hormones, transcription factors, microRNAs, and chromatin-modifying proteins as being also involved in the establishment of male and female traits in a hermaphroditic flower (reviewed in Ó'Maoiléidigh et al., 2014; Chávez Montes et al., 2015). Therefore, the processes during stamen and carpel differentiation require the interplay of extensive regulatory networks, that are directly or indirectly related to the floral organ identity genes (Wellmer et al., 2004; Zhang et al., 2005). Hence, organ abortion or loss-of-function in unisexual flowers in different species may involve a mutation or differential regulation in any of the many regulatory genes that control **androecium** or **gynoecium** differentiation, in what probably reflects the multiple evolutionary origins of dioecy and monoecy (Ainsworth, 2000). Diggle et al. (2011) summarized the literature on unisexual flower development, and recognized four stages of sexual organ loss of organ function: before the initiation of stamen or carpel primordia (stage 0); early in stamen or carpel development (stage 1); pre-meiosis (stage 2); and post-meiosis (stage 3) (Figure 1). Among the 292 taxa surveyed by these authors, loss-of-sexual-organ function occurs with equal frequency at each of the four stages in both male and female flowers, in monoecious and dioecious taxa. The arrest of development does not tend to occur preferentially at any particular stage, suggesting that there are no key stages of androecial or gynoecial development that are affected repeatedly upon the evolutionary origin of unisexual flowers. Therefore, a direct comparison of the pathways responsible for the establishment of unisexual flowers in different species is likely to reveal conserved modules of gene regulatory hubs involved in stamen or carpel development, as well as differences that reflect the different stages of development in which male and/or female organ arrest or loss-of-function occur.

## *Quercus suber:* a case study

With the aim of uncovering the developmental programs underlying the establishment of female and male organs in unisexual flowers of a monoecious species, a recent study published in *Frontiers of Plant Science 2014, 5:599* has identified differentially expressed genes during the development of male and female flowers of *Quercus suber* (cork oak), an ecologically and economically important Mediterranean tree.

**Figure 2:**
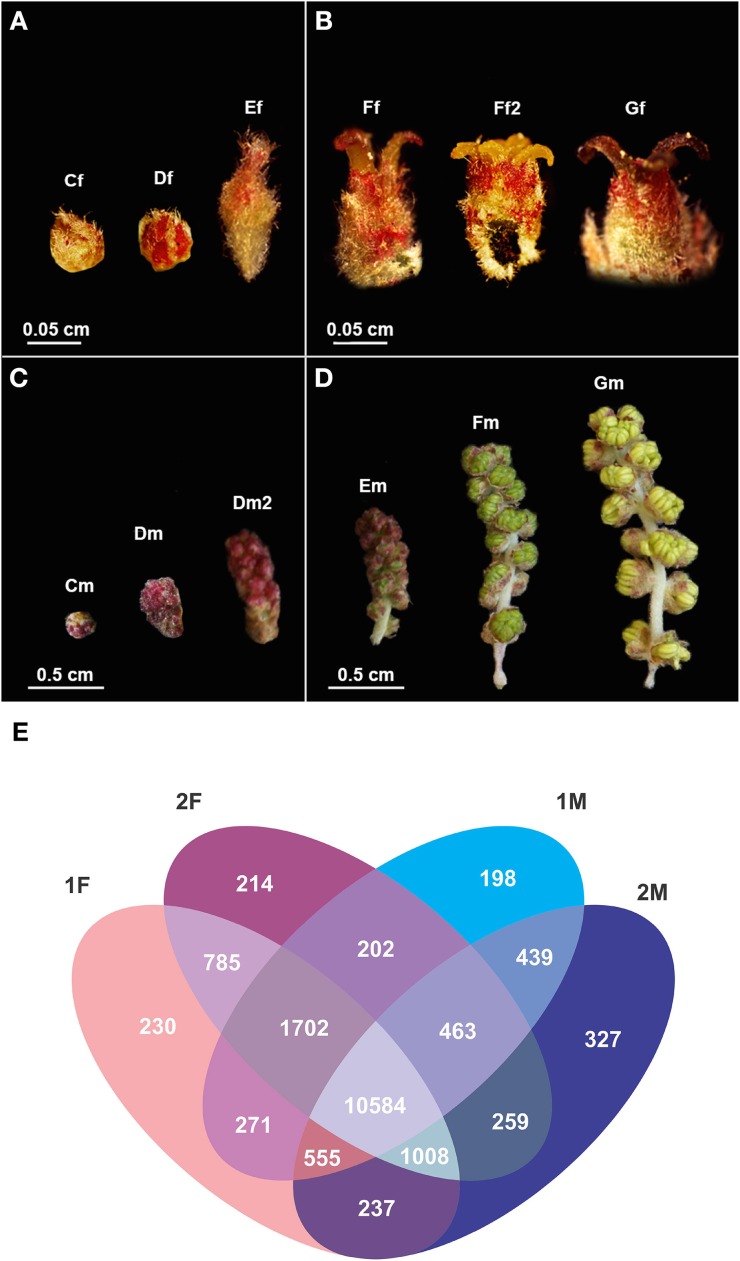
***Quercus suber* female and male flowers in different developmental stages used in the RNA-seq and a description of the unique and differentially expressed gene between libraries. (A)** Early and **(B)** late stages of female flower development used in pools 1F and 2F, respectively. **(C)** Early and **(D)** late stages of male flower development used in pools 1M and 2M, respectively. (Cf) female bud enclosed by protective scales; (Df) female reddish bud with open scales; (Ef) elongation of the spike axis and the emergency of the first pair of flowers; (Ff) female flower showing distinct, erect, yellow stigmas with curved pinkish/brownish tips; (Ff2) flower with shining yellow and viscous pattern stigmas in clear divergent position; (Gf) female flower with closed stigmas that lost the receptivity, exhibiting a dark brown color. (Cm) catkin with red round shape due to the tight clustering of the flowers; (Dm) elongated cluster of male flowers; (Dm2) pendent catkin with some individualized flowers; (Em) male flowers with the anthers individualized; (Fm) flowers with individualized green/yellow anthers where pollen shedding begins; (Gm) catkin with male flowers in full anthesis. **(E)** Venn diagram indicating the number of exclusive and shared transcripts of early and late developmental stages of *Quercus suber* flower. Four EST projects were generated from four-specific RNA pools, two for female flowers (1F and 2F) and two for male flowers (1M and 2M), covering either early (1F and 1M) or the late (2F and 2M) developmental stages (Rocheta et al., 2014).

In *Q. suber*, female inflorescences arise in spikes, with three to five individual flowers (Figures 2A,B), on the axil of the new leaves. Female flowers are included in a cupule and contain three carpels, with two ovules each (Boavida et al., 1999). Male flowers are organized in catkins that emerge in reproductive buds of the previous growth season or at the base of the branches of the current season. Each individual catkin contains 15–25 staminate flowers (Figures 2C,D). The staminate flowers present a perianth with four to six tepals with an equal or double number of anthers that do not burst simultaneously (Boavida et al., 1999). *Q. suber* flowers are thought to be unisexual by inception, as there is no morphological evidence of organ initiation or abortion of the missing organs (Varela and Valdiviesso, 1996; Boavida et al., 1999). Interestingly, *Q. suber* is a **protandrous** species, with male catkins developing in early spring and sometimes also in autumn, whereas female flowers appear in spring, more than a month later than the male ones, and only get fully developed a few months later, if pollinated. Therefore, this species presents spatial separation of male and female reproductive organs, but by delaying the maturation of the carpels, it presents also a temporal separation, as there is little overlap between staminate and pistillate phases of an individual plant. This was previously referred to as “temporal dioecism” (Cruden and Hermann-Parker, 1977) and it exists in many species as a means to achieve outbreeding. Another characteristic trait of this species is its long **progamic phase**. At the time of pollination, the ovary is still undifferentiated and the transmitting tissue extends only to the base of the styles. Usually, the pollen tube germinates and its growth is arrested for 6 weeks at the base of the style, overlapping with ovule differentiation (Boavida et al., 1999; Kanazashi and Kanazashi, 2003). Therefore, to capture the complete development of the female flower up to the maturation of the ovules, the tissues have to be sampled up to 6 weeks after pollination.

KEY CONCEPT 3 **Protandrous system and progamic phase**

A **protandrous system** can be defined as a sexual strategy in which the development of male flowers occurs before the development of the female flowers. A **progamic phase** is the period that spans between pollination and fertilization.

Using an RNA-seq approach, Rocheta et al. (2014) generated distinct cDNA libraries for early and late stages of male and female flower development of *Q. suber* providing a high-throughput study on female and male flowers of this monoecious tree, hence, within a uniform genetic background. In this study, six developmental stages of male and female cork oak flowers were included (Figures 2A–D) to cover early stages of organ development (1M and 1F), up to pollen release in male flower and pollination in female flowers (2M and 2F, respectively). As it is difficult to track which female flowers are successfully pollinated and, hence, proceed to complete the differentiation of the embryonic sac, these stages of female flower development after pollination were not sampled. This *Q. suber* transcriptomic study revealed differential accumulation of transcripts in male and female flowers and in different stages of flower development (Figure 2E). The analysis of the cDNA libraries obtained showed that there were 230 unique contigs for the early (1F) and 214 contigs unique for the late (2F) stages of female flower development. The 1F unique contigs might correspond to genes controlling early flower development, whereas the 2F unique contigs might be associated with stigma maturation. Accordingly, there were 198 contigs unique in the early stages of male flower development (1M), most probably involved in early stages of anther development and 327 contigs specific for the late stages (2M) that could be indicative of genes controlling pollen development and maturation. Further analysis showed that the majority of the unique transcripts in the male libraries are organ specific, not having been detected in other organs of the plant, and probably reflect the uniqueness of the stamen regulatory network. On the contrary, most of the transcripts found over-represented in the female libraries are also expressed in other organs (root, leaves, buds, and fruits).

Diggle et al. (2011) stated that, in unisexual flowers that do not initiate the undesired organs, the likely sex-determinant genes should be among the factors in the short pathway from floral initiation to organ identity establishment, particularly the B and C class organ-determinacy genes and their regulatory effectors. Studies in species that are dioecious by inception, *Spinacea olearacea* and *Thralictrum dioicum*, or are monoecious by early organ abortion, *Elias guineensis*, are clear examples that demonstrate how changes in the regulation of homeotic regulators can initiate different sex-developmental pathways (Jaligot et al., 2004; Adam et al., 2005; Sather et al., 2010; Larue et al., 2013). In *S. oleracea*, B class genes are expressed before the initiation of floral organ primordia in a sex-specific manner and its suppression in male flowers by RNAi originates a conversion of male into female flowers (Sather et al., 2010). Similar results were obtained in *T. dioicum*, where targeted silencing of a B class gene (*TdPI*) by virus-induced gene silencing also resulted in homeotic conversion of stamens into carpels (Larue et al., 2013). The oil palm *Elaeis guineensis* has staminate unisexual flowers by inception and pistillate flowers that contain a pair of aborted stamens (Adam et al., 2005). An *E. guineensis* floral variant known as *mantled*, commonly observed in palms produced by *in vitro* micropropagation is characterized by the homeotic transformation of the fertile or sterile androecium into pseudocarpels resembling B class mutants (Jaligot et al., 2004; Adam et al., 2005). The *mantled* phenotype was associated with the DNA hypomethylation of a LINE retrotransposon (*Karma*) present in the intron of the B class gene *DEFICIENS* leading to alternative splicing and premature transcript termination (Ong-Abdullah et al., 2015).

In *Q. suber*, the three B-class transcripts (*QsAPETALA3, QsPISTILLATA*, and *QsTM6*) were more abundant in the male flower libraries, whereas *QsAGAMOUS* (the C-class organ identity gene) had a similar level of expression in male and female libraries (Rocheta et al., 2014). Down-regulation of the B-class genes may explain the absence of stamens during pistillate flower development. It would be interesting to address the function of the B-class genes in *Q. suber* in future studies, such as the analysis of their expression domain within the male flower meristem, how their transcripts are temporally and spatially regulated in young male and female flower primordia and if these genes are under epigenetic regulation that could lead to differential expression between the different flower types. There are few high-throughput transcriptomic studies comparing male and female flowers by inception. An example is a study made available by Song et al. (2013a) that performed a microarray analysis on male and hermaphrodite flowers of an andromonoecious poplar in a late stage of development prior to pollination (male organs of male flowers were compared to the female organs of the hermaphrodite flowers). These authors acknowledged the advantages of studying different flower ontogenic processes on the same plant, thus having the same genetic background. The transcription profile of several genes in poplar was very similar to the one observed in *Q. suber* for late stages of flower development (e.g., *QsAGL24* over-represented in the male organs and *QsCOL9* in the female ones). It would be interesting to investigate if in early stages of organ development there is a similar transcription profile between *Q. suber, P. tomentosa, S. olearacea*, and *T. dioicum.* The data provided by Rocheta et al. (2014) might be important to complement future studies in other trees species and, therefore, could create an opportunity to uncover genes that are involved in reproductive biology of species in which sex differentiation occurs before or during the reproductive organ primordia initiation.

## Transcriptomic analysis of unisexual flowers in other species

Successful functional studies in a non-model tree species, such as cork oak, are difficult to perform, due to several limitations that are transversal to many non-model species: a lack of a sequenced genome, a recalcitrant behavior to transformation and a long life cycle, and for many years not much was known about the transcription profiling that underlie their developmental programs.

The recent availability of high-throughput technologies has allowed the generation of large-scale data in non-model species. Comparative global transcriptome analysis between studies may reveal conserved gene regulatory modules involved in the differentiation of male and female organs that underlie the development of unisexual flowers. However, to make the most of transcriptomic studies, a comparison between studies should take into account the type of biological material utilized, in particular, the chosen developmental stages used for RNA extraction, the sexual strategy of each species (e.g., monoecy or dioecy), the type of developmental events that establish unisexuality (unisexual by inception or by organ abortion), and the type of high-throughput platform and the pipeline of bioinformatic data treatment (Table 1).

**Table 1:** **Recent transcriptomic studies with species exhibiting unisexual flowering**.

**Species**	**Sexual system**	**Stage of dimorphism emergence**	**Description**	**Organs compared**	**Method**	**References**
*Quercus suber*	Monoecious	Before flower organ primordia initiation	Transcriptomic profiling between early and late stages of male and female flower development	Early and late stages of female and male flower development	454 technology	Rocheta et al., 2014
*Populus tomentosa*	Dioecious	Before flower organ primordia initiation	Transcriptomic analysis of male and female flowers of an andromonoecious population	Last phase of flower development, before pollination (produced in cosexual catkins)	Agilent Genechip Poplar Genome Array	Song et al., 2013a
			Transcriptomic analysis of microRNAs of flowers from an andromonoecious population		Illumina technology	Song et al., 2013b
*Cucumis sativus*	Monoecious	Stamen development arrested after the differentiation between the anther and filament. Pistil arrest occurs before the differentiation between the stigma and ovary (Bai et al., 2004)	Transcriptomic differences between flowers from a gynoecious and hermaphrodite populations	Hermaphrodite and female flower buds with approximately 5 mm in diameter, a critical stage for sex determination (Bai et al., 2004)	454 technology	Guo et al., 2010
			Transcriptomic differences between gynoecious and monoecious populations	Apices from plants at 2.5 leaf stage	Illumina technology	Wu et al., 2010
*Cucumis melo*	Monoecious	Similar to *Cucumis sativus* (Boualem et al., 2008)	Transcriptomic differences between gynoecious, hermaphrodite, andromonoecious, and monoecious populations	Pool of apices from four stages: true leaf at the 2-true-leaf stage (before transplanting), axillary buds and fresh leaves (1 week after transplanting), flower buds (< 2 mm in length), and flower buds before flowering	Illumina technology	Gao et al., 2015
*Zea mays*	Monoecious	The arrest of stamens and pistil occurs early during organ growth in pistillate (ear) florets, staminate (tassel) florets (Le Roux and Kellogg, 1999)	Transcriptomic analysis of the tassel and ear at different developmental stage	Female: pre-emergence cob; post-emergence cob, silk, and ovule. Male: pre-emergence tassel, post-emergence tassel, whole anthers, and pollen. Seed: 5 days after pollination (DAP) and 10 DAP. Embryo: 25 DAP and endosperm: 25 DAP	Illumina technology	Davidson et al., 2011
			Transcriptomic analysis of the ear at different developmental stage	Ears at four developmental stages: the growth point elongation, spikelet differentiation, floret primordium differentiation, and the floret organ differentiation phases	Illumina technology	Liu et al., 2015
*Silene latifolia*	Dioecious	Anthers are arrested in growth soon after they emerge, prior to the internal structures formation. In male flowers, a thin filament that bears little resemblance to normal carpels develops in an abnormal primordium (Grant et al., 1994)	Comparison between transcriptomes of male and female plants, focusing in X- and Y-linked alleles	Buds at developmental stages B1–B2, after removing the calyx	Illumina technology	Muyle et al., 2012
				Flower bud tissues	Illumina technology	Bergero and Charlesworth, 2011
				Entire shoots with flower buds of male and female plants	Illumina technology	Chibalina and Filatov, 2011
*Diospyros lotus*	Dioecious	Female flowers generally cannot produce pollen grains and male flowers have a residual carpel (Akagi et al., 2014)	Transcriptomic differences between male and female flowers. Reads mapped to the MSY-linked region	Early differentiation stages of male/female primordia	Illumina technology	Akagi et al., 2014
*Carica papaya*	Trioecious	Female flowers have no traces of stamens and male flowers have a reduced ovary (Ronse Decraene and Smets, 1999)	Transcriptomic differences between male, female, and hermaphrodite individuals, focusing in the sex-chromosome-specific tags	Male, female, and hermaphrodite flowers in early (7 mm) and late (20 mm) stages	Ht-Super SAGE	Urasaki et al., 2012
*Asparagus officinalis*	Dioecious	Stamen arrest occurs in the onset of meiosis. Pistil abortion may occurs at various stages after meiosis (Caporali et al., 1994)	Transcriptomic differences between male, female, and supermale plants	Spear tips	Illumina technology	Harkess et al., 2015
*Vitis vinifera*	Hermaphrodite (cultivated population) and dioecious (wild population)	In male flowers ovule development is not inhibited, but the style and stigma are missing. The suppression of maleness appears to be the consequence of pollen sterility (Dorsey, 1912, 1914; Caporali et al., 2003)	Comparison between male, female and hermaphrodite flower transcriptomes of hermaphrodite and dioecious populations	Flower bud in the stages B, D, G, and H of male, female, and hermaphrodite plants Developmental stages according to Baggiolini (1952)	Illumina technology	Ramos et al., 2014
*Salix suchowensis*	Dioecious	Not available	Transcriptomic differences between male and female flowers RNA-seq of reads mapped to the MSY-linked region	Flower buds expanded but unflushed without the bud bracts	454 technology	Liu et al., 2013
*Prunus mume Sieb. et Zucc*	Hermaphrodite with imperfect (staminate) flowers phenomenon	The staminate flowers are characterized by either pistils below the stamens, withered pistils, or an absence of pistils (Hou et al., 2011)	Transcriptomic analysis of small RNAs in perfect vs. staminate flowers	Hermaphrodite and staminate flowers developing at early December, when the pistils of imperfect flowers stop differentiating	Illumina technology	Gao et al., 2012
			Transcriptomic analysis of differentially expressed genes in perfect vs. staminate flowers		Illumina technology	Shi et al., 2012

The type of sexual system, the stage of dimorphism emergence, the tissues collected for RNA extraction, and the platform utilized for data collection are indicated.

In the past decade, several high-throughput transcriptomic studies comparing male and female flowering structures from different species have been made available, and to which the results obtained by Rocheta et al. (2014) may be compared against. An initial survey of the available transcriptomic studies (see references within Table 1) reveals that some authors are particularly interested in addressing the regulation of potential candidates within certain groups of genes, particularly, hormone-related genes, genes linked to sexual chromosomes, transcriptional regulatory factors, miRNAs, and epigenetic modifiers. Other authors describe modules of genes that are differentially co-expressed and may represent a particular transcriptional network active in a specific stage of flower organ development.

## Hormonal regulation

Hormones play wide-ranging roles in the development and physiological processes throughout the lifetime of an angiosperm plant from seed to senescence. In the past century several authors have emphasized the influence of hormonal regulation in sex determination (e.g., Rudich et al., 1972) but an overall mechanism linking hormone signaling and sex determination has not been possible mainly due to the ambiguous action of hormones, which in many cases is species dependent. In *Q. suber*, the great majority of the genes associated with auxin, ethylene, and cytokinin regulatory pathways are over-represented in the female flowers. In opposition, several gibberellins-related transcripts were found exclusively in the male flowers, being expressed both in early and late stages of development, indicating a role in male floral primordia and in anther differentiation (Rocheta et al., 2014). The masculinization role of gibberellins (Cheng et al., 2004; Plackett et al., 2011; Zhang et al., 2014) is, however, not consensual as genes promoting ear development in maize are associated with the gibberellin biosynthesis pathway (Bensen et al., 1995; Winkler and Helentjaris, 1995; Helliwell et al., 2001). The same happens with ethylene, as in species such as maize, cucumber, or melon, the feminizing role of ethylene appears to be conserved (Boualem et al., 2008; Eveland et al., 2010; Sun et al., 2010; Liu et al., 2015), whereas in *Citrullus lanatus* ethylene has a masculinizing effect (Rudich, 1990). Despite a lack of a universal consensual role in flower masculinization or feminization for each particular hormone, in the different transcriptomic studies surveyed, groups of hormone-related transcripts have been identified as being differentially expressed between flower types, which suggests a definite role for hormones in sexual organ differentiation and should be seen as species specific (Guo et al., 2010; Wu et al., 2010; Song et al., 2013a; Ramos et al., 2014; Rocheta et al., 2014; Gao et al., 2015).

## Transcriptional regulation

Differential regulation of transcription factors (TF) has a pivotal role in the control of mechanisms that control organ development (Latchman, 1997). There are several examples of how individual transcription factors can control sex developmental pathways and organ differentiation in unisexual species (Chuck et al., 1998, 2007; Martin et al., 2009). Taking into consideration the functional importance of TF, Rocheta et al. (2014) described several unique and differentially expressed TF between the male and female libraries and grouped them in families according to their homologies. Other high-throughput studies followed the same reasoning and pointed out amongst the differential expressed genes in each study several transcription factors that could be determinant for sex differentiation (e.g., Guo et al., 2010; Wu et al., 2010; Huang et al., 2013; Gao et al., 2015; Liu et al., 2015). In the Cucurbitaceae, sex-determination genes associated to the ethylene biosynthesis pathway are partly conserved (Yamasaki et al., 2001; Mibus and Tatlioglu, 2004; Knopf and Trebitsh, 2006; Martin et al., 2009; Li et al., 2012) but little is known about the transcriptional machinery associated to sex determination. To fill up this gap, Wu et al. (2010), Guo et al. (2010), and Gao et al. (2015) conducted RNA-seq studies in the genus cucumber using similar strategies. In *C. sativus*, male flowers from monoecious individuals were compared to female flowers from gynoecious individuals, whereas in *C. melo* a broader range of genotypes were used (Gao et al., 2015). The number of differential expressed genes varied depending on the study, maybe reflecting different sequencing platforms or different methods used for data analysis. Using *454* pyrosequencing technology, Guo et al. (2010) identified 90 genes up regulated in female flowers and 124 genes in the bisexual flower, whereas Wu et al. (2010) using *Solexa* technology identified a greater amount of differentially expressed genes (143 up-regulated and 600 down-regulated in female flowers). Several identified homologs for transcription factors (*BEL-1 LIKE HOMEODOMAIN1, PHYTOCHROME INTERACTING PROTEIN3, WRKY, MYC2*) were also differentially expressed in the *Q. suber* libraries (Rocheta et al., 2014). Gao et al. (2015) performed a transcriptomic survey of melon inflorescences from individuals with different sexual habits (monoecious, andromonoecious, hermaphrodite, and gynoecious). Using *Illumina* technology, four flower libraries were obtained for each genotype and further analysis allowed the identification of genes unique to each library. Particularly, a homolog for *PIF3* that is exclusive to female flowers (similarly to *Q. suber*) was identified. *PIF3* codes for a protein that in Arabidopsis interacts with DELLA proteins (Feng et al., 2008) and, thus, also suggests the involvement of gibberellins in sex determination in cucumber. The TFs common to the aforementioned studies were zinc fingers that have been previously associated with the masculinization of *C. melo* flowers (Martin et al., 2009). Interestingly, all the zinc fingers differentially expressed in *Q. suber* are over represented in the female libraries (Rocheta et al., 2014). Regarding the Cucurbitaceae, it would be very interesting to analyze the data using a similar pipeline in order to identify common transcriptional regulation networks.

Several high-throughput transcriptomic studies have been performed to study the reproductive development of maize (Wang et al., 2009; Zhu et al., 2009; Eveland et al., 2010; Davidson et al., 2011; Kakumanu et al., 2012; Sekhon et al., 2013; Liu et al., 2015). A recent RNA-seq study analyzed solely the formation of the female flower (Liu et al., 2015). Four developmental stages were analyzed (Table 1) that ranged from the undetermined meristem to floret organ differentiation. Several differentially expressed genes were identified in pairwise comparative analysis between the different stages of development (Table 1), including a group of transcription factors whose expression in specific stages could be crucial for female flower development. Davidson et al. (2011) compiled an extensive transcriptomic data on several stages of the maize reproductive development. The study included four male libraries (pre- and post-emergence tassel, whole anthers and pollen) and four female libraries (pre- and post-emergence ear, ear, and ovule). More than identifying specific candidate genes associated with sex differentiation, the study identified not only transcripts unique to each developmental stage but also modules of genes that have similar patterns of expression that may be involved in common regulatory mechanisms. It would be very interesting to compare the libraries from both studies (Li et al., 2015; Davidson et al., 2011) in order to find a common signature for the female flower differentiation program.

Differential regulation of miRNAs and epigenetic modifications have emerged as potential sex determinants (Parkinson et al., 2007; Martin et al., 2009; Song et al., 2015). Epigenetic mechanisms involved in sex determination were mentioned in melon (Martin et al., 2009) but have been also described in other species bearing unisexual flowers. In maize, the factor *Rmr6* maintain repressed epigenetic stages and, consequently, the monoecious sexual system of maize is preserved by limiting the function of the pistil-protecting factor, *SILKLESS1*, from the apical inflorescence (Parkinson et al., 2007). Deep sequencing of male and female flowers of an andromonoecious poplar allowed the identification of more than 100 miRNAs differentially expressed between the male and female libraries with at least five miRNAs targeting transcripts located in the *P. tricocharpa* sex chromosome, that are involved in several plant development processes from disease resistance to hormonal regulation (Song et al., 2013b). It is worth mentioning that some miRNAs target epigenetic regulators, and are, in turn, regulated by epigenetic modifications, suggesting a feedback between different mechanisms in the control of sex differentiation (Song et al., 2015, 2013b). Another study used the imperfect flowers that naturally occur in the Japanese apricot (*Prunus mume*) to identify several miRNAs that are unique and associated specifically to pistil development (Gao et al., 2012; Shi et al., 2012).

## Environmental control of unisexual flower development

In *Q. suber*, the emergence of male and female flowers are spatially and temporally separated, with the female flowers emerging at least a month later than the male flowers. In some years, a flush of male flowering is observed again in late summer. On the other hand, the spatial distribution of *Q. suber* flowers is somehow difficult to predict as, on the same tree, not all the branches exhibit flowers and there are years that only one type of flowers (or none at all) develop. Therefore, it is possible that the induction of these unisexual flowers is under environmental control and might be mediated by epigenetic regulation.

The effect of environmental factors on flower sex ratio in plants has long been the subject of studies of many scientists (Harper, 1907), and some proposed that the environment may influence the evolution of single factor sex-determining systems (reviewed in Charlesworth, 2013). Many other authors have tried to analyze how flower-organ developmental pathways are under the influence of abiotic and biotic factors (e.g., Stehlik et al., 2008). For instance, sex determination in the monoecious oil palm is strongly influenced by environmental factors (reviewed in Adam et al., 2011) and dioecious *S. oleraceae* plants grown under water restriction displayed a male-biased sex ratio (Freeman and Vitale, 1985). Day length and light intensity also affect sex ratio in plant populations. *Atriplex halimus* (Chenopodiaceae), a monoecious species (and sometimes polygamous), displays increased femaleness under short days and low-light irradiance (Talamali et al., 2003). Therefore, the susceptibility of some plant species to alter their sex-developmental pathways in response to external stimuli suggests that there is a strong interaction between the environmental parameters and genetic regulatory mechanisms. It is possible that small environmental changes such as abiotic stress, day length or temperature may alter epigenetic marks to generate a particular flower sex phenotype. The link between environmental signals, DNA methylation or other epigenetic signatures and plant sex determination has not yet been established, but future studies should explore this further.

## What lies ahead?

The suppression of male and female functions in flowering plants has evolved more than 1000 times (reviewed in Renner, 2014) giving rise to unisexual flowering systems that promote outbreeding and are considered a driving force in plant evolution. However, the development of a unisexual flower has several biological implications, some of which may culminate in failure to produce progeny. Several economically important species have unisexual flowers (e.g., *Actinidia deliciosa, Carica papaya, Diospyros lotus*) and that poses a problem for producers with several reports suggesting low profitability due to short number of female flowers and consequent decrease in seed production (e.g., *Jatropha curcas*). Similarly, the production of acorns in *Q. suber* is difficult to predict because in some years an individual tree might only develop one type of unisexual flower. Thus, the identification of female- and male-determining pathways that trigger the formation of unisexual flowers within the same or in different individuals is an important step to maximize agricultural returns.

Several genetic studies have provided a framework in which several genes were identified as part of sex-determining pathways in distant related species. For instance, recently, a genetic model for sex determination in *C. melo* and *C. sativus*, integrating genes that control ethylene kinetics, shed light in how male and female flowers coexist, and how the ratio of male to female flower can be modulated in the same plant in these Cucurbitaceae species (Boualem et al., 2015). However, for other species, such *as Q. suber*, a long life cycle makes it difficult the use of genetics to uncover mechanisms of flower gender-determination. The advent of high-throughput technologies, and the generation of large-scale data in non-model species, was a turning point in uncovering potential genes involved in unisexual flower development. However, in most of these studies, the identification of specific genes involved in sex determination was not achieved. Some reasons for this may be that, for most flower tissues being utilized, sex determination has already occurred (Table 1), or that the developmental stages reflecting real sex-determining switch points have not been sampled. Therefore, a description of the biological samples sequenced and the stages of organ abortion in each species must be really well documented and taken into consideration in the experimental design.

The independent origin of unisexual flowers across angiosperms along with the varied developmental pathways and stages to achieve unisexuality guarantees that there will be no universal genetic program that regulates unisexual flower determination. However, transcriptomic studies have been providing valuable information on the genes and pathways that are differentially involved in male and female flower development for each species, and provide an excellent platform for future functional research. Most of these studies generate a list of genes that are differentially expressed between libraries and then only pinpoint some genes amongst these that have previously reported functions in the same or in other species. This approach leaves out many genes potentially important and thorough unbiased analysis should provide many more new genes involved in the process. Future studies should try to integrate direct comparison of libraries from different studies (those reviewed in Table 1 and others), especially if using phylogenetically close species, or species with similar stages of flower organ abortion, which will uncover potential similar processes involved in sex-determining switch points, as well as downstream, tissue-specific modules regulating male and female flower organ developmental pathways.

The transcriptomic data of early and late stages of male and female flower development of *Q. suber* provides a new tool for studies of unisexual flower development by inception. Temporal and spatial separation of male and female flowers in this species enabled the identification of genes potentially involved in the differentiation of each flower type within the same genetic background. Several questions are still open for discussion namely the mechanisms associated with the induction of each type of flowers at different time points during the reproductive season. The cork oak susceptibility to alter its sex-developmental pathways at different times during the year suggests that the induction of these unisexual flowers is under environmental control, as observed in other species such as spinach (dioecious; Freeman and Vitale, 1985), oil palm or *Atriplex halimus* (monoecious; reviewed in Talamali et al., 2003; Adam et al., 2011). However, it is unclear how the environmental factors influence the triggering of specific sex-determining pathways. As the activity of genes is capable of being modified in response to environmental changes by epigenetic control (Turner, 2009), the relationship between environmental signals, DNA methylation and plant organ determination must be addressed.

Moreover, future studies toward the understanding of specific flower organ determining pathways should involve not only the generation of databases based the transcriptomic data for each species flower tissues with the integration of potential known regulatory networks but also be combined with molecular biology experiments (e.g., *in situ* hybridization, yeast-one-hybrid, yeast-two-hybrid, epigenetic analysis) performed during and preferentially before the first signs of organ differentiation (or abortion) in order to predict sex-determination related mechanisms.

## Author contributions

All authors listed, have made substantial, direct and intellectual contribution to the work, and approved it for publication.

### Conflict of interest statement

The authors declare that the research was conducted in the absence of any commercial or financial relationships that could be construed as a potential conflict of interest.
